# Chronic O-GlcNAcylation and Diabetic Cardiomyopathy: The Bitterness of Glucose

**DOI:** 10.3389/fendo.2018.00642

**Published:** 2018-10-29

**Authors:** Simon Ducheix, Jocelyne Magré, Bertrand Cariou, Xavier Prieur

**Affiliations:** ^1^l'institut du thorax, INSERM, CNRS, UNIV Nantes, Nantes, France; ^2^l'institut du thorax, INSERM, CNRS, UNIV Nantes, CHU Nantes, Nantes, France

**Keywords:** glucotoxicity, diabetes, metabolism, cardiomyopathy, O-GlcNAcylation

## Abstract

Type 2 diabetes (T2D) is a major risk factor for heart failure. Diabetic cardiomyopathy (DC) is characterized by diastolic dysfunction and left ventricular hypertrophy. Epidemiological data suggest that hyperglycaemia contributes to the development of DC. Several cellular pathways have been implicated in the deleterious effects of high glucose concentrations in the heart: oxidative stress, accumulation of advanced glycation end products (AGE), and chronic hexosamine biosynthetic pathway (HBP) activation. In the present review, we focus on the effect of chronic activation of the HBP on diabetic heart function. The HBP supplies N-acetylglucosamine moiety (O-GlcNAc) that is O-linked by O-GlcNAc transferase (OGT) to proteins on serine or threonine residues. This post-translational protein modification modulates the activity of the targeted proteins. In the heart, acute activation of the HBP in response to ischaemia-reperfusion injury appears to be protective. Conversely, chronic activation of the HBP in the diabetic heart affects Ca^2+^ handling, contractile properties, and mitochondrial function and promotes stress signaling, such as left ventricular hypertrophy and endoplasmic reticulum stress. Many studies have shown that O-GlcNAc impairs the function of key protein targets involved in these pathways, such as phospholamban, calmodulin kinase II, troponin I, and FOXO1. The data show that excessive O-GlcNAcylation is a major trigger of the glucotoxic events that affect heart function under chronic hyperglycaemia. Supporting this finding, pharmacological or genetic inhibition of the HBP in the diabetic heart improves heart function. In addition, the SGLT2 inhibitor dapagliflozin, a glucose lowering agent, has recently been shown to lower cardiac HBP in a lipodystophic T2D mice model and to concomitantly improve the diastolic dysfunction of these mice. Therefore, targeting cardiac-excessive O-GlcNAcylation or specific target proteins represents a potential therapeutic option to treat glucotoxicity in the diabetic heart.

## Introduction

The incidence of heart failure (HF) is 2.5-fold higher in patients with type 2 diabetes (T2D) than in healthy subjects (1). Although the number of myocardial infarctions has been reduced by 25% in the T2D population over the last 10 years, HF is becoming the chronic diabetic complication of greatest concern (2). Ischaemic heart disease and hypertension are associated with HF in 65 and 75% patients with T2D, respectively (3). However, a fraction of patients with T2D display HF without ischaemic injuries; this observation has led to the identification of specific diabetes-related cardiomyopathy (DC) (4). DC is characterized by diastolic dysfunction and left ventricular hypertrophy (5, 6). In a large cohort study, Iribarren and colleagues demonstrated that for each 1% increase in glycated hemoglobin (HbA1C), the HF-related death or hospitalization rate increased by 8% (7). A recent study confirmed that HF-related hospitalization is more frequent in patients with higher HbA1c levels (8). These clinical observations suggest that hyperglycaemia *per se* contributes to the development of DC.

Indeed, a large body of evidence supports that altered cardiac energetic substrate utilization and metabolic inflexibility contribute to DC physiopathology (5, 6). The diabetic heart is characterized by an insulin resistance that compromises glucose uptake and metabolism (9), resulting in an increased reliance on lipids (10) and leading to increased fatty acid uptake (11) and ectopic lipid accumulation in cardiomyocytes (12). In the diabetic heart, pyruvate dehydrogenase (PDH) activity is inhibited by the accumulation of acetyl-CoA as a result of elevated fatty acid catabolism via the ß-oxidation pathway (5). In addition, insulin resistance directly inhibits the activity of phosphofructokinase-2, which regulates glycolysis rate (9). In heart of *ob/ob* insulin-resistant mice, insulin is unable to stimulate glucose oxidation (13). Overall, the diabetic heart is characterized by a glucose overload (14). Cellular studies have demonstrated that high glucose activates apoptosis in cardiomyocytes (15), thereby leading to the development of the concept of glucotoxicity.

Several cellular pathways are suspected to mediate the deleterious effects of high-glucose concentrations in the heart: oxidative stress, accumulation of advanced glycation end-products (AGEs), and chronic hexosamine biosynthetic pathway (HBP) activation [for a review, see (16)]. Hyperglycaemia can feed the pentose phosphate pathway that produces NADPH from glucose-6-phosphate (17). NADPH is the substrate of the cytosolic NADPH oxidase, an enzymatic complex that generates reactive oxygen species (ROS) (18). Therefore, hyperglycaemia contributes to ROS production and eventually oxidative stress, which can affect cardiac function. In addition, high glucose levels favor the non-enzymatic glycation reaction that produces AGEs (19). In the context of diabetes, AGEs have been shown to impair the function of glycated proteins, modify the properties of extra-cellular matrix, and activate RAGE, the AGE receptor that induces ROS production and thus contributes to oxidative stress (20). Furthermore, chronic activation of the HBP has been extensively studied in the diabetic heart. The HBP supplies UDP-N-acetylglucosamine moiety (UDP-GlcNAc) that is O-linked by O-GlcNAc transferase (OGT) to proteins on serine or threonine residues (21). This post-translational protein modification modulates the activity of the targeted proteins and has been described in different organisms and organs as a cellular stress response (22). UDP-GlcNAc can also be N-linked to asparagine residues and other HBP intermediary products and can display biological activity independently of the O-GlcNAcylation process. However, in this review, we focus on the effects of the general HBP pathway and the O-GlcNAcylation process in the diabetic heart due to the greater knowledge of these phenomena than of UDP-GlcNAc-related processes.

In the heart, HBP is increased in response to ischaemia/reperfusion and appears to be protective by limiting cytosolic calcium entry (23, 24); it is also increased during trauma hemorrhage (25, 26). After ischaemia/reperfusion, OGT over-expression promotes cell survival and attenuates oxidative stress and calcium overload (27, 28). A large body of work suggests that pharmacological or genetic activation of the HBP is beneficial for post-ischaemic function [for reviews, see (22, 29)]. In contrast, cardio-specific and inducible deletion of OGT exacerbates cardiac dysfunction after ischaemic/reperfusion injury (30). However, as with most stress-response pathways, although acute induction is often protective, chronic activation might be deleterious. In this report, we review how chronic HBP activation contributes to the deleterious effects of glucose overload in the diabetic heart. We review the specific actors of the HBP and discuss therapeutic interest in targeting this pathway for developing pharmaceutical approaches to treat DC.

## Chronic activation of the HBP in the diabetic heart

Early studies demonstrated that chronic activation of the HBP is associated with the development of insulin resistance in adipose tissue (31) and skeletal muscle (32). While the enzymatic activities of the rate-limiting enzyme of the HBP, glutamine: fructose-6-phosphate amino-transferase (GFAT), and of O-GlcNAc transferase (OGT) are present in most insulin-sensitive tissues, OGT activity has been found to be particularly elevated in the heart (33). As hyperglycaemia was suspected to be involved in the cardiac dysfunction associated with diabetes, Ren et al. tested the effects of elevated glucose concentration on cardiomyocyte properties (34). Using neonatal cardiomyocytes, these researchers demonstrated that high-glucose exposure induces excitation-contraction (E-C) coupling impairment and that this induction was mimicked by glucosamine treatment but not by treatment with non-metabolically active glucose analogs. These results suggested that glucose metabolism intervenes in this induction effect and, more precisely, that HBP chronic activation might lead to cardiomyocyte dysfunction. The effects observed with glucosamine treatment suggested that HBP activation not only is correlated with cardiomyocyte dysfunction but is also able to compromise E-C coupling. Further investigating the HBP pathway, Pang et al. confirmed that hyperglycaemia modifies the calcium entry in neonatal cardiomyocytes and that this modification was reversed by treatment with azaserine, an inhibitor of GFAT (35). In accordance with a role of HBP in cardiac function, Clark et al. demonstrated that calcium cycling alteration was associated with an increase in the abundance of total O-GlcNAcylated protein levels in neonatal cardiomyocytes (36). *Ex vivo*, in heart isolated from streptozotocin (STZ)-treated mice, a mouse model of type 1 diabetes (T1D), phenylephrine-induced inotropy was blunted, and this blunting was correlated with UDP-GlcNAc accumulation (37).

Supporting these *in vitro* and *ex vivo* observations, O-GlcNAcylated protein accumulation was first found in STZ-treated mice (38). Similarly, in most common mouse models of T2D, cardiac dysfunction has been associated with increased levels of O-GlcNAcylated protein (39–42). Importantly, O-GlcNAcylated protein levels are increased in cardiac biopsies isolated from human patients with heart failure (43).

Recently, we performed cardiac phenotyping of seipin KO (SKO) mice, a model for generalized lipodystrophy, a rare genetic disease characterized by a near absence of adipose tissue, insulin resistance, and T2D (44). SKO mice display a DC phenotype with diastolic dysfunction and left ventricular hypertrophy (LVH) that correlate with hyperglycaemia. The most hyperglycaemic mice display the more severe cardiac phenotype. The heart of SKO mice displays glucose overload evidenced by strong induction of the HBP. In contrast, we did not identify any lipotoxic hallmark in the heart of SKO, nor did we observe ROS production, AGE accumulation, or fibrosis. To the best of our knowledge, this report describes the first mouse model of DC in which chronic HBP activation is the only glucose-overload hallmark present. It can be hypothesized that excessive O-GlcNAcylation alone can trigger the cardiac abnormalities associated with hyperglycaemia in T2D.

Altogether, *in vitro* and *in vivo* evidence strongly indicates that chronic HBP activation is associated with cardiac dysfunction in diabetes. This observation raises the question of causality: How do elevated O-GlcNAcylated protein levels affect cardiac function?

## Chronic HBP and cardiac contractile properties

The diabetic heart is characterized by impairments of the contractile and electrophysiological properties, which might be mainly due to defects in calcium handling and myofilament function. These two parameters are intrinsically linked, as myofilament properties define calcium sensitivity (${\text{ECa}}_{50}^{2+}$), an indication of myofilament strength production at the basal calcium level. As previously mentioned, high-glucose exposure was initially associated *in vitro* with HBP activation and E-C impairment (34). This E-C impairment is reversed by OGT inhibition (35). Therefore, several research groups have aimed to understand how chronic HBP activation alters calcium handling and, in turn, E-C in cardiomyocytes. As cardiac-type sarcoplasmic reticulum Ca^2+^ ATPase (SERCA2) is central in calcium handling in the cardiomyocyte, Yokoe et al. tested the ability of O-GlcNAcylation to modulate its activity (45). Following the treatment of cardiomyocytes with an inhibitor of OGA (PUGNAC) to increase the levels of O-GlcNAcylation, they did not observe an increase in the levels of O-GlcNAcylated SERCA2 but did observe an increase in phospholamban O-GlcNAc levels. This finding suggests that *in vitro*, HBP induction itself prevents normal Ca^2+^ pumping after excitation. Phospholamban regulates SERCA2 activity: In its unphosphorylated state, it inhibits SERCA2 activity, whereas it induces SERCA2 activity when phosphorylated on Ser16. In STZ-induced diabetic mice heart, an opposite pattern of regulation of O-GlcNAcylation on the phosphorylation of phospholamban on Ser16 was reported, with elevated O-GlcNAc levels leading to reduced phospholamban phosphorylation. Such increased O-GlcNAcylation of Ser-16 and its reciprocal decrease in phosphorylation leading to depressed SERCA2 activity was also observed in isolated cardiomyocytes. The *in vivo* relevance of the reciprocal post-translational modification of phospholamban has been established in a mouse model of T2D, in which mice display progressive ser16-phosphorylation decrease and O-GlcNAcylation increase concomitant with the appearance of diastolic dysfunction (40).

Another key actor in heart calcium homeostasis that is regulated by O-GlcNAcylation is calmodulin-dependent protein kinase II (CAMKII). Erickson et al. showed that high glucose induced CAMK activity in isolated cardiomyocytes and that this induction was repressed by pharmacological inhibition of OGT (46). These researchers identified an O-GlcNAc site on CAMKII and later demonstrated that CAMKII activation increases the frequency of the Ca^2+^ spark. The levels of O-GlcNAc-CAMKII measured in cardiac biopsies from T2D/HF patients have been found to be markedly elevated relative to the levels of non-diabetic controls. This finding is in agreement with the potential deleterious effect of chronic CAMKII activation in HF. In addition to regulating the ER Ca^2+^ store, the capacitative calcium entry (CCE) pathway controls Ca^2+^ entry in the cell. In neonatal cardiomyocytes, hyperglycaemia exposure inhibits CCE, which is reversed by azaserine, thereby directly involving the HBP (35). O-GlcNAcylation of STIM1, an ER protein involved in CCE, prevents its interaction with the plasma membrane and in turn disturbs CCE (47). However, O-GlcNAc levels of STIM1 have not yet been evaluated in diabetic heart. Altogether, these data indicate that phospholamban, CAMKII and STIM1 activity are modified by chronic HBP activation, thereby altering Ca^2+^ homeostasis in the diabetic heart.

Hypothesizing that calcium handling alone cannot account for the contractile dysfunction observed in the diabetic heart, Ramirez-Correa et al. highlighted the potential role of myofilament proteins (48). In an initial study, they identified O-GlcNAc sites in actin and troponin 1 and showed that O-GlcNAcylation decreased myofilament sensitivity to Ca^2+^ and consistently increased ECa^2+^ 50. The O-GlcNAcylated levels of several contractile proteins, including myosin heavy chain, actin, and tropomyosin, are increased in STZ-treated mice heart (48, 49). These increases in O-GlcNAcylation levels are concomitant with changes in OGT/OGA activity, and, interestingly, diabetes is associated with a sarcomeric re-localization of OGT and OGA. Although in control heart, OGT and OGA are mainly located at the Z-line and the A-band, respectively, this distribution is greatly altered in the diabetic heart, raising the hypothesis that the OGT/OGA activity ratio is partially determined by their sub-cellular localization (49). Interestingly, removing GlcNAc residues by recombinant OGA restores the ECa^2+^ 50 in cardiac skinned fibers isolated from T1D heart. Therefore, chronic HBP activation alters the activity of proteins involved in Ca^2+^ handling and directly controls the contractile properties of the Ca^2+^-sensitive myofilament component, thus regulating cardiac contraction and relaxation (49).

In addition to diastolic dysfunction and contractile properties, prolonged QT is associated with increased risk of ventricular arrhythmias in the diabetic heart. In the heart of STZ mice, increased O-GlcNAc-modified NAV1.5 levels have been associated with increased arrhythmia scores, suggesting that HBP chronic activation may directly affect cardiomyocyte electrical activity (50).

## HBP chronic activation promotes hypertrophy signaling

Left ventricular hypertrophy (LVH) is a common feature in cardiac dysfunction and is found in several pathophysiological contexts. In cardiac conditions such as aortic stenosis, aortic banding and myocardial infection, LVH was found to be positively correlated with elevated levels of O-GlcNAc protein, suggesting that chronic HBP activation may lead to LVH (43). Interestingly, both chronic glucose overload in the heart and aorta banding lead to increased UDP-N-acetylglucosamine, which has been associated with LVH (51). In both metabolic and physical models, LVH results in a similar pressure overload-induced hypertrophy pattern involving increased expression of ßMHC, the fetal isoform of MHC (51). Transcription factors NFAT (52), GATA4, and MEF2C (53) induce a cardiac hypertrophy gene expression program, e.g., inducing atrial (ANP) and B-type (BNP) natriuretic peptide mRNA expression, following their activation by O-GlcNAcylation. Consistent with the direct induction of HBP, pharmacological inhibition of GFAT in isolated cardiomyocytes prevents the mRNA induction of ANP and BNP under phenylephrin hypertrophic signaling. These findings clearly indicate that O-GlcNAcylation can induce hypertrophy through regulating gene expression; however, the direct involvement of this mechanism *in vivo* in the context of diabetes has not yet been described. Ding et al. aimed to identify the mechanism involved in cardiac hypertrophy in the diabetic heart (54) and found that in isolated cardiomyocytes, high-glucose exposure induced increases in cardiomyocyte size and ANP, BNP and ßMHC mRNA levels. This hypertrophic pattern was associated with elevated HBP activity and was reversed by HBP inhibition (55). The authors proposed that the HBP-induced hypertrophy was due to ERK1/2 and Cyclin D2 activation that paralleled the O-GlcNAcylation increase. In diabetic STZ rats, elevated O-GlcNAc levels have similarly been associated with a hypertrophic profile (of cardiomyocyte size and gene expression) and the induction of the ERK1/2 pathway (54). This work further suggests the involvement of O-GlcNAc in diabetic-associated LVH but does not explain how O-GlcNAcylation modulates the ERK1/2 pathway. Cyclin D2 activation by the transcription factor c-MYC has also been found to be involved in cardiac hypertrophy (56). Interestingly, c-MYC has an O-GlcNAcylation site (55), but the effect of its O-GlcNAcylation in the diabetic heart remains unknown.

Although NFAT and ERK1/2 activation have been found to be involved in O-GlcNAcylation-mediated hypertrophy, recent studies indicate that the relationships are complex. Gelinas et al. have shown that AMPK activation counteracts cardiomyocyte hypertrophy both *in vitro* and *in vivo* (57). Unexpectedly, AMPK activation did not prevent NFAT or ERK1/2 hypertrophic-associated induction but was required to lower HBP activity. Furthermore, AMPK activation led to the normalization of troponin T O-GlcNAcylation levels: Troponin T O-GlcNAcylated levels were elevated in the hypertrophic heart and reduced after AMPK activation. Previous work reported that in heart failure, increased troponin T O-GlcNAcylation was concomitant with reduced phosphorylation levels (58). Therefore, troponin T is potentially an important target of the HBP in hypertrophic heart or heart failure; however, to date, no relevant data are available for the diabetic heart. Further work is needed to determine whether troponin T O-GlcNAcylation is elevated in the diabetic heart.

In addition to its effects in cardiomyocytes, high-glucose exposure increases O-GlcNAcylation of the transcription factor Sp1 in rat cardiac fibroblasts. Chromatin immunoprecipitation experiments demonstrated that SP1 O-GlcNAcylation increases its ability to bind collagen 1 promoter and, in turn, to increase its expression, thereby contributing to hypertrophy-associated fibrosis (59).

## Chronic HBP activation impairs mitochondrial function

The diabetic heart displays an altered energetic substrate utilization profile, and mitochondrial dysfunction appears central in this feature. Notably, cardiac fibers isolated from patients with T2D but not those from obese patients display a disturbances in the mechanical properties associated with mitochondrial dysfunction (60). In rat neonatal cardiomyocytes, high-glucose exposure induces an increase in mitochondrial protein O-GlcNAc levels, which is reversed by OGA overexpression (61). Both mitochondrial complex I subunit NDUFA9 and the complex IV component COXI display elevated O-GlcNAc levels. Consistently, complex I and complex IV activities, O_2_ consumption and ATP production were reduced in high-glucose exposed cardiomyocytes, and these reductions were reversed by OGA overexpression. These findings were confirmed in hearts from insulin-resistant rats, in which O-GlcNAcylation of NDUFA9, COXI, and VDAC, the voltage dependent anion channel, was increased (62). Mitochondrial activity is also regulated by the dynamic balance between fusion and fission. In cardiomyocytes, O-GlcNAcylation has been shown to increase the function of the mitochondrial fission regulator, DRP1 (63). Importantly, increased O-GlcNAcylated DRP1 levels are associated with mitochondrial fragmentation, which leads to reduced mitochondria activity. Such increases in DRP1 O-GlcNAcylated levels have also been observed *in vivo* in diabetic rat hearts.

Excessive mitochondrial O-GlcNAcylation was recently shown to compromise mitochondria integrity. In STZ-treated mice, 8-oxoguanine DNA glycosylase (OGG1), a key enzyme in the mitochondrial DNA repair machinery, is modulated by O-GlcNAcylation (64). Although *Ogg1* expression is induced in diabetic hearts, its enzymatic activity is blunted. Using *in vitro* and *in vivo* approaches, researchers demonstrated that O-GlcNAcylation of OGG1 decreased its activity. Furthermore, these researchers showed that *in vivo* inhibition of O-GlcNAcylation improved mtDNA repair in the heart of STZ-treated mice (64).

Overall, growing evidence suggests that mitochondria are major sites for O-GlcNAcylation. Banerjee et al. revealed the dynamics of mitochondrial branching and hydrolysis of UDP-GlcNAc as well as its transport through the mitochondrial inner membrane in the T1D rat heart. The authors also showed that diabetes affects the mitochondrial levels of OGA and OGT and their localization and activity. Moreover, they identified that UDP-GlcNAc was actively imported within mitochondrial matrix through Pyrymidine Nucleotide Carrier (Pnc1). Furthermore, they modulated OGA and OGT in neonatal rat cardiomyocytes and showed that OGA but not OGT inhibition reduced ATP production and the oxygen consumption rate (65). In addition, a general proteomic analysis of cardiac mitochondria isolated from STZ-treated rats revealed that numerous proteins involved in many and varied biochemical processes such as pyruvate decarboxylation, fatty acid (CPT1) and Ca^2+^ transport, fatty acid oxidation, electron transport chain processes, and oxidative scavenging undergo the O-GlcNAc process (66). Identifying the precise role of each O-GlcNAcylation (for some proteins at different sites) is of interest to decipher the contribution of mitochondrial O-GlcNAc in diabetes-associated cardiac dysfunction.

## ER stress, autophagy and the HBP

Unresolved activation of the unfolded protein response (UPR), i.e., the endoplasmic reticulum (ER) stress response, has been observed in several metabolic complications, such as liver steatosis and insulin resistance (67, 68). Regarding DC, elevated ER stress has been reported (69, 70), but its exact contribution to DC physiopathology is not clear. After ischaemia/reperfusion injury, Wang et al. observed concomitant increases in key players involved in the HBP and UPR, such as spliced X-Box Binding Protein 1 (XBP1), the chaperone BIP and the pro-apoptotic transcription factor CHOP in heart lysates. At the mechanistic level, they demonstrated that XBP1 binds the GFAT1 promoter and activates its expression, but they did not observe an increase in XBP1 O-GlcNAcylation (71). Acute UPR activation appears to be protective during the acute induction of HBP by ischaemia/reperfusion. Further work is required to determine whether a common mechanism is activated in DC. It would be interesting to test whether ER stress induction can, in addition to hyperglycaemia, contribute to the chronic induction of HBP.

Several studies have observed a cross-talk between the UPR and autophagy that involves several key players, such as the elongation factor eIF2A (72–74). Autophagy is a cell process in which unused or defective cellular materials, such as unused/defective macromolecules or organelles, are degraded and recycled. It occurs via a complex pathway, the regulation of which is finely controlled by nutrient availability and hormonal status, that involves a high number of genes. In the heart, autophagy is associated with aging and several cardiac pathologies, such as myocardial infarction and ischaemia/reperfusion injury, cardiac hypertrophy, cardiotoxicity and diabetic cardiomyopathies [for a recent review, see (75)]. Regarding type 1 and type 2 diabetes-associated cardiomyopathies, the role of autophagy remains debated. In T1D animal models, autophagy has been described to be either protective (76–78) or deleterious in the heart (78). Autophagy is repressed in the heart of different T2D mice models (76, 79, 80), where its induction appears beneficial (76). However, other studies have reported unchanged and elevated levels of myocardial autophagy in HFD models (81) and fructose-induced insulin resistance (82), respectively. Autophagy is also upregulated in the heart of patients with T2D (83). Such different levels of autophagy observed in both T1D and T2D might be explained by the variation in the models used; additional studies are needed to decipher whether autophagy is a beneficial or a deleterious process in the diabetic heart. Nevertheless, autophagy was shown to be blunted in cardiomyocytes isolated from *db/db* mice, and this blunting was reversed by HBP inhibition (80). Interestingly, in the hepatocyte cell-line HepG2, autophagy induction by mTOR inhibition resulted in HBP inhibition, further suggesting a link between these two pathways. Taken together, several elements suggest potential cross-talk among ER stress, autophagy and HBP, but their relationships in the context of DC warrant further investigation.

## Targeting excessive O-GlcNAcylation as a therapeutic strategy

**Figure 1:**
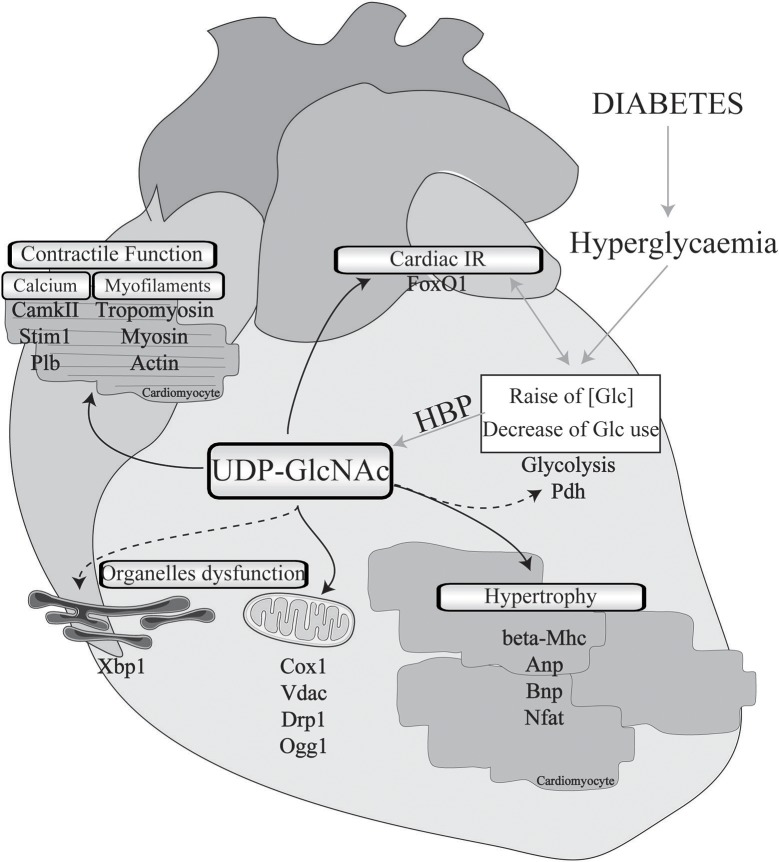
Implication of O-GlcNAcylation in diabetes-associated cardiomyopathies. Cardiac physio-pathological processes that have been shown to be affected by O-GlcNAc during diabetes are highlighted with black arrows. Pathways or proteins that have been shown to be modulated by O-GlcNAc in non-diabetic cardiac injury or in other organs with a diabetic background are indicated with black dotted arrows. IR, insulin resistance; Glc, glucose; HBP, hexosamine biosynthesis pathway; Pdh, pyruvate dehydrogenase; Mhc, myosin, heavy polypeptide; Anp, natriuretic peptide type A; Bnp, natriuretic peptide type B; Nfat, nuclear factor of activated T-cells; Cox1, cytochrome c oxydase subunit I; Vdac, voltage-dependent anion channel; Drp1, dynamin-related protein 1; Ogg1, 8-oxoguanine DNA-glycosylase 1; Xbp1, X-box binding protein 1; CamkII, calcium/calmodulin-dependent protein kinase II; Stim1, stromal interaction molecule 1; Plb, phospholamban; FoxO1, forkhead box O1.

Data from the literature strongly suggest that excessive O-GlcNAcylation during chronic HBP activation affects Ca^2+^ handling, contractile properties, and mitochondrial function and promotes stress signaling, such as that involved in hypertrophy and ER stress (impacted cardiac physio-pathological processes and proteins are highlighted in Figure 1 and Table 1). Therefore, excessive O-GlcNAcylation might be considered an innovative target to treat cardiac dysfunction associated with diabetes. Interestingly, in most of the above-described experiments with isolated cardiomyocytes, pharmacological inhibition of GFAT or OGT was used to modulate the HBP. Pharmacological modulation of the HBP *in vivo* is more challenging. The first proof of concept that targeting the HBP is beneficial was achieved in STZ mice with cardiac overexpression of OGA: The reduction in O-GlcNAcylated protein levels concomitantly improved the contractile properties (38). At the mechanistic level, the adenoviral vector-mediated OGA overexpression was shown to improve Ca^2+^ handling and to restore phospholamban phosphorylation levels. Such a beneficial effect on Ca^2+^ handling was confirmed in T2D mice, where inducible overexpression of OGA led to improvements in the LV properties assessed by normalization of the fractional shortening (40). Another way to modulate OGA expression is to use a microRNA-539 inhibitor (84). In a post-myocardial infarction HF mouse model, OGA mRNA levels were found to be reduced in association with the induction of miRNA-539. In neonatal cardiomyocytes, the overexpression of miRNA-539 suppressed OGA expression and consequently increased general O-GlcNAc levels, whereas an miRNA-539 inhibitor rescued OGA protein expression and restored O-GlcNAcylation (84). It would be very interesting to study anti-miR-539 in a T2D mouse model and its effects on the HBP and cardiac function.

**Table 1:** O-GlcNAcylated targets in the diabetic heart.

**Protein**	**O-GlcNAcylation**	**Physiological effect**	**Effect on activity**
Phospholamban	Yes-S16	E-C alteration	Decrease
CAMKII	Yes-S279	Increased Ca2+ spark frequency	Increase
MHC	Yes-S844, S1471, S1472, T1601, S1917	Reduced myofilament calcium sensitivity	Decrease
Actin	Yes-T326	Reduced myofilament calcium sensitivity	Decrease
aTropomyosin	Yes-S87	Reduced myofilament calcium sensitivity	Decrease
Nav1.5	Yes	Arrhythmia	Decrease
NFAT	No	Hypertrophy	Increase
GATA4	Yes	Hypertrophy	Increase
MEF2C	Yes	Hypertrophy	Increase
SP1	Yes	Collagen over-production	Increase
NDUFA9	Yes	Reduced mitochondria activity	Decrease
COXI	Yes	Reduced mitochondria activity	Decrease
VDAC	Yes	Reduced mitochondria activity	Decrease
CPT1B	Yes S180	Reduced mitochondria activity	Decrease
DRP1	Yes T585, T586	Fragmentation/Reduced mitochondria activity	Increase
OGG1	Yes	Alteration of mitochondria integrity	Decrease
FOXO1	Yes	Association with insulin resistance	?

*These proteins display elevated O-GlcNAc level in the diabetic heart. The table indicates whether formal demonstration of O-GlcNAcylation (IP/WB of the O-GlcNAc form or spectrometry) has been made, and, when known, the O-GlcNAc sites. The physiological effects of elevated O-GlcNAc levels in DC and the effects on target activity are listed*.

Genetic deletions of OGT have also been used to modulate the HBP. As total KO for OGT is lethal, Watson et al. used constitutive cardio-specific deletion of OGT. They reported only 12% survival and the surviving animals displayed dilated cardiomyopathy and heart failure (reduced ejection fraction and cardiac output) (85). The same research group developed an inducible cardio-specific deletion of OGT (30, 85), but to our knowledge, this model has not been applied in the context of DC. Such an investigation is crucial as a proof-of-concept test evaluating whether OGT alone is a suitable target.

In addition to the effects of direct HBP modulation on cardiac dysfunction, the effects of other therapeutic interventions on cardiac O-GlcNAc levels highlight the potential therapeutic value of modulating the HBP. As mentioned earlier, in a mouse model of angiotensin II-induced cardiac hypertrophy, AMPK activation exerts its beneficial effect on the heart by reducing HBP, especially troponin T-O-GlcNAc levels (57). Importantly, these effects are lost under treatment with NbutGt, an OGA inhibitor, which indicates that the effects are specific and O-GlcNAc dependent. Again, it would be interesting to conduct similar experiments in T2D mouse models.

As mentioned earlier, in our mouse model of congenital generalized lipodytrophy, we hypothesized that glucotoxicity is central to the cardiac phenotype. Treatment of those mice with dapagliflozin, an SGLT2 inhibitor, normalized glycaemia and, concomitantly, O-GlcNAc protein levels and cardiac function (44). These results are encouraging, although a formal demonstration that HBP normalization contributes to the cardiac benefits has yet to be made.

It has been shown that exercise training mitigates chronic activation of the HBP in type 1 diabetic rats. Two months of daily swim training improved rat heart rate and normalized the protein levels of the calcium pump SERCA2. These improvements were associated with a reduction in OGT activity, a decrease in total O-GlcNAcylated protein levels and a reduction in O-GlcNAcylated Sp1 level (86). These findings raise the hypothesis that the beneficial effect of training on cardiac function might be, at least partially, attributable to an effect of exercise on the HBP. These interesting finding have been challenged by another report showing that exercise increased the general levels of O-GlcNAcylation in *db/db* mice (87). Further studies are needed to describe the potential benefits of exercise on cardiac HBP chronic activation in diabetes.

## Future directions and conclusions

Fifteen years ago, chronic activation of the HBP was described as a hallmark of glucose overload in the diabetic heart. In the following years, excessive O-GlcNAcylation has been shown to impair calcium handling and contractile properties, promote hypertrophy, and compromise mitochondrial functions. Several direct targets of O-GlcNAcylation, including phospholamban, CAMKII and troponin I, appear to play central roles in the deleterious effect of HBP chronic activation. Interestingly, O-GlcNAc often occurs at the same or nearby Ser/Thr amino acid residues as phosphorylation, and competition may exist between the two phenomena to control the activity of important players in heart activity and metabolism. Such interplay has been extensively reviewed previously (88, 89).

Despite the fact that DC is associated with a state of insulin resistance, few studies have highlighted the effect of the HBP on insulin resistance. Whereas, O-GlcNAcylation of several insulin-signaling key players (such as AKT, PT1B, and PDK1) has been shown to be associated with insulin resistance in the liver (90–93), the effect of the HBP on insulin signaling in the heart has not been adequately addressed. Recently, we reported that FOXO1 was O-GlcNAcylated in the heart of lipodystrophic diabetic mice and that SGLT2i treatment reversed this increase (44). O-GlcNAcylation of FOXO1 increases its activity. FOXO1 is believed to be a key mediator of glucotoxicity in different organs (94). Notably, it has been found to be involved in metabolically induced cardiac dysfunction, especially insulin resistance. FOXO1 knock-down was shown to be protective in a model of diet-induced cardiomyopathy (95, 96). Our work revealed that FOXO1 O-GlcNAcylation is associated with heart insulin resistance and cardiac dysfunction. In our study, the levels of O-GlcNAcylated AKT2 appeared unchanged. Further studies are needed to explore whether FOXO1 O-GlcNAcylation is a common hallmark in DC, whether it occurs in other heart samples from T2D models, and whether other insulin-signaling players are O-GlcNAcylated in DC. The question that needs to be addressed is whether O-GlcNAcylation is solely a mediator of diabetic associated glucotoxicity in DC or whether it also disturbs insulin sensitivity and thus promotes cardiac insulin resistance?

As chronic HBP activation occurs in DC concomitantly with other metabolic abnormalities such as lipotoxicity, ROS production, AGE accumulation and other cardiac pathological manifestations such as fibrosis, it is difficult to determine its direct effects on cardiac function. *In vitro* studies with glucosamine or PUGNAC (OGA inhibitor) treatment have indicated that the HBP not only is correlated with cardiomyocyte dysfunction under chronic hyperglycaemia but can also induce associated abnormalities (e.g., in E-C coupling and contractile properties). However, *in vivo* evidence that chronic HBP can by itself cause DC remains weak, and to the best of our knowledge, there are no publications showing the effect of OGT overexpression or OGA deletion in the heart *in vivo*. Nonetheless, several attempts to suppress the HBP in DC, either by pharmacological approaches, mainly *in vitro*, or by genetic overexpression *in vivo*, suggest that excessive O-GlcNAcylation alone might trigger DC. In addition, in SKO mice, we reported DC associated only with HBP chronic activation, i.e., with HBP chronic activation in absence of any other DC hallmark. Altogether, these observations raise the question of whether the HBP can be modulated as a therapeutic target to treat DC. Several issues should be addressed. First, the HBP is a very balanced pathway, and the modulation of OGT might induce a feedback loop by OGA and *vice versa*. This possibility suggests moderate modulation be attempted. Second, the HBP is a very broad pathway that operates in all tissues; thus, the question of tissue specificity should be addressed. Finally, although pharmacological interventions have been used *in vitro*, they have not been widely used *in vivo*. Genetic interventions seem to be the most suitable way to modulate the HBP. For these reasons, a targeted approach could be considered. Consideration should be given to the possibility of targeting the O-GlcNAcylation of specific proteins in specific tissues, which would require the identification of HBP targets in the diabetic heart.

## Author contributions

XP wrote the manuscript and supervise the work between the authors. SD, JM, and BC wrote the manuscript.

### Conflict of interest statement

The authors declare that the research was conducted in the absence of any commercial or financial relationships that could be construed as a potential conflict of interest.
